# Adolescence, Violence, and Public Health

**DOI:** 10.3389/fpubh.2013.00032

**Published:** 2013-09-17

**Authors:** Joav Merrick, Isack Kandel, Hatim A. Omar

**Affiliations:** ^1^National Institute of Child Health and Human Development, Jerusalem, Israel; ^2^Health Services, Division for Intellectual and Developmental Disabilities, Ministry of Social Affairs and Social Services, Jerusalem, Israel; ^3^Division of Pediatrics, Hadassah Hebrew University Medical Center, Jerusalem, Israel; ^4^Division of Adolescent Medicine, Kentucky Children’s Hospital, University of Kentucky, Lexington, KY, USA

**Keywords:** public health, child health, adolescence, violence, homicide, suicide, school violence, home visitation

## Introduction

Adolescent or youth violence is a very visible violence in our modern society, where you just have to open the newspaper or the television and you find yourself right in the middle of it. In order to understand the scope of the problem, we need to look at the epidemiology of global violence. In 1996 the World Health Assembly declared violence a leading public health issue (1) and as a result of this resolution a comprehensive report was published in 2002 (2).

The World Health Organization (WHO) defined violence as: “The intentional use of physical force or power, threatened or actual, against oneself, another person, or against a group or community, that either results in or has a high likelihood of resulting in injury, death, psychological harm, maldevelopment, or deprivation” (3).

The WHO’s Global Burden of Disease project for 2000 (2) estimated that 1.6 million people (28.8 per 100,000) worldwide died as a result of self-inflicted, interpersonal, or collective violence. In this 2000 project an estimated 199,000 adolescent homicides (9.2 per 100,000) occurred globally or about 565 adolescents died each day due to interpersonal violence with variations around the globe. Homicide rates were 0.9 per 100,000 in countries like Europe, 17.6 per 100,000 in Africa, and 36.4 per 100,000 in Latin America (2). So we indeed live in a violent world, but there has been a downward trend between 1990 to 2000 from a violence related death rate of 35.3 to 28.8 per 100,000 (2, 4). Each year, more than 1.6 million people worldwide lose their lives to violence (5). For every person who dies as a result of violence, many more are injured and suffer from a range of physical, sexual, reproductive, and mental health problems. Violence places a massive burden on national economies, costing countries billions of US dollars each year in health care, law enforcement, and lost productivity (5).

## Trends in Adolescent Homicide

In the years between 1985 and 1994 the adolescent homicide rates increased in many parts of the world. There was a sharp increase for males (it doubled), while the trend for females was steady. The increase was seen in developing countries and economies in transition with the most common method of attack by firearm. Dramatic increase was seen in the age group of 10- to 24-year-olds in the Russian Federation after the collapse of communism with a 150% increase from 7.0 to 18.0% per 100,000. Decrease was seen in Canada and Australia, while both the United States and New Zealand had increase (2).

Between 1970 the early 1990s, the homicide rate for teens ages 15 to 19 more than doubled, from 8.1 to a peak of 20.7 per 100,000 in 1993 (6). The rate declined steeply during the late 1990s, then leveled out at around nine deaths per 100,000 between 2000 and 2004. Although the rate of homicides increased between 2004 and 2006, to 10.7 deaths per 100,000, it has since decreased. In 2010, the homicide rate was 8.3 deaths per 100,000, the lowest it has been since before 1980 (7).

Risk factors can be divided into four categories (8): family factors such as a firearm at home, low income, domestic violence, teen parents, or divorce; social factors such as ethnic heterogeneity, crowded housing, racial intolerance, lack of adult supervision, and social acceptance of violence; psychological factors such as depression, antisocial behavior, conduct disorder, or aggression; and personal factors such as male gender, alcohol or drug abuse, poor impulse control, previous gunshot injury, and minority race.

## Trends in Adolescent Suicide

Suicide is the third leading cause of death among adolescents with a fivefold increase seen for example in the United States for the period 1950–1990 (8). In 2010, rates of suicide among male teens were highest among American Indians (24.3 per 100,000) and whites (14.2), followed by Hispanics at 8.1, blacks at 6.8, and Asian or Pacific Islanders at 6.3 per 100,000 (7). Among females, American Indian teens had the highest rate at 11.0 per 100,000, followed by white teens at 3.5, Hispanic teens at 2.9, and Asian or Pacific Islanders with 3.1, with black teens at 1.1 per 100,000 (7).

Firearms and alcohol are also here important risk factors (4–7) and gender. Adolescent females attempt more suicide, but males have a higher completion rate due to the use of more lethal means (like a firearm).

## Trends in Non-Fatal Violence

For every adolescent homicide there is about 20–40 victims of non-fatal youth violence (2). Here still males are most often involved, but fewer firearm involvement and instead fists and feet and other weapons like knife and clubs (2).

## Trends in School Violence

Research into school violence took off in Scandinavia and the United kingdom in the 1980s and together with the WHO cross national studies (37 countries) on health behavior in school aged children (HBSC) is conducted every fourth year (9). Bullying in schools varied from 15.7% in Sweden to 68.5% in Lithuania and with 50.0% in Israel (9).

According to the CDC’s School Associated Violent Death Study, between 1 and 2% of all homicides among school-age children happen on school grounds or on the way to and from school or during a school sponsored event (10). In a 2011 nationally representative sample of youth in grades 9–12 (11):

- 12% reported being in a physical fight on school property in the 12 months preceding the survey
- 16% of male students and 7.8% of female students reported being in a physical fight on school property in the 12 months preceding the survey
- 5.9% did not go to school on one or more days in the 30 days preceding the survey because they felt unsafe at school or on their way to or from school
- 5.4% reported carrying a weapon (gun, knife, or club) on school property on one or more days in the 30 days preceding the survey
- 7.4% reported being threatened or injured with a weapon on school property one or more times in the 12 months preceding the survey.

## Examples from Our Daily Practice

We would like to share a few stories from our with adolescents on a daily basis:

- Two 13-year-old boys were arrested for breaking into air-conditioning units in the neighborhood to steal Freon. Obviously they use it for huffing, to get high. Upon interviewing them after being consulted by the social services, one of the boys said: “I am bored, my parents are working and I have nothing to do after school so we wanted to get high and forget about life’s problems”
- A young man of 14 years of age was admitted after attempting suicide. He said his life was basically good, but he became worried that maybe he is not a real man, since he noticed that his breasts are growing and he is looking more like a girl
- Several young teens between the ages of 13–15 years were diagnosed with gonorrhea tonsillitis. They said they were not sexually active, but have participated in “head parties” with other peers. It was revealed that these parties referred to gathering of young teens to have oral sex.
- A 16-year-old girl was referred for evaluation of depression and weight loss. She disclosed that she had been sexually assaulted by her stepfather for the last 2 months. When we tried to line up her mother’s help we got exactly the opposite: the mother threatened to kick the “liar” out of the home without even listening to the whole story.

We also see our fair share of good stories with teens doing good deeds and achieving high levels of success. It is the bad stories, however, that grab our attention, because of their sad and tragic consequences and our desire to understand why they happen. In countries like the United States of America the leading causes of mortality continue to be accidents, homicide, suicide, sexually transmitted infections, and teen pregnancy. All are preventable yet they continue to happen. So whom should we blame? The parents for being busy and/or uneducated, the media for portraying sex, drugs, and violence as a “normal” way of life, the school system for not educating our kids about real life, the health care industry that continues to deprive many of our teens access to health care, or our local and federal leaders both political and religious for giving bad examples with their continuing scandals of corruption and inappropriate sexual conduct!

It would be extremely simplistic to say that one or the other bears the blame. With the technological advances and the availability of modern communication systems including the internet, our teens are bombarded with a sea of information that is frequently inaccurate or age-inappropriate. The literature and real life experience are overall in agreement that improvement of all aspects of our societies is needed to help prevent many of our teens’ problems. The parents should do their part in finding a way to spend quality time with their kids and to educate them on all aspects of life. Schools should do a better job of teaching real life skills and knowledge rather than purely academic subjects only. As a society, we should be able to provide better environment and health care for our adolescents. Our leaders should be providing good examples and so should the celebrities. If each one of us contributes, maybe we can improve our future by improving the current status of our adolescents who are our future.

## Home Visitation

One type of prevention programs that has proven effective is home visitation. Such programs dates back nearly 100 years in Scandinavia, the United Kingdom, and even in the United States with research showing the positive long-term effects (12). Prenatal and early childhood nurse home visitation (2 years after birth) can reduce serious antisocial and criminal behavior shown by a 15-year closely follow-up study (12).

## Conclusion

In recent years a growing body of research on adolescent violence has shown the epidemiology on a global basis and confirmed that we are talking about a major public health problem. Programs for intervention has also been researched and long-term follow-up (12) indicates that early intervention is working. Such programs would seem to demand a substantial economic investment, but proven cost-effective in the long run (13).
